# Thyroid Storm Presenting With Neurological, Obstetric, and Cardiovascular Features: A Case Series

**DOI:** 10.7759/cureus.106859

**Published:** 2026-04-11

**Authors:** Daniel Balderrama

**Affiliations:** 1 Internal Medicine, Hospital Metropolitano, Monterrey, MEX

**Keywords:** adult seizures, atrial fibrillation, case series, graves' disease (gd), ischemic stroke, thyroid storm

## Abstract

Thyroid storm is a rare but life-threatening endocrine emergency that can present with a wide range of clinical manifestations, which may delay diagnosis, particularly in atypical cases. We present a case series of three patients with thyroid storm to highlight its clinical variability and management challenges. We describe three patients with thyroid storm who presented with distinct clinical patterns, including neurological involvement with seizures, an obstetric trigger with severe systemic deterioration, and a case complicated by atrial fibrillation and ischemic stroke. In all cases, a precipitating factor was identified, and treatment was initiated with beta-blockers, corticosteroids, and methimazole. Although scoring systems such as the Burch-Wartofsky Point Scale were useful, they did not always fully reflect clinical severity, particularly in the presence of comorbid conditions. All patients showed clinical improvement with treatment. These cases illustrate the clinical variability of thyroid storm and the importance of clinical judgment in diagnosis. They also support the use of methimazole as a reasonable therapeutic option, within the limitations of this case series, in settings where propylthiouracil is not available.

## Introduction

Thyroid storm is generally considered the most severe form of thyrotoxicosis and, although uncommon, remains a critical condition with a significant risk of mortality if not recognized early [1,2]. In clinical practice, one of the main challenges is that disease severity does not always correlate with the degree of hormonal elevation, but rather with the patient’s systemic response to excess thyroid hormone [1,3].

Thyroid storm is associated with significant mortality, particularly when diagnosis and treatment are delayed [1,2].

The mechanisms underlying this exaggerated response are not fully understood but likely involve increased sensitivity to catecholamines and enhanced peripheral conversion of thyroid hormone [1]. Most patients have an identifiable precipitating factor, with infection being the most common, followed by surgery, trauma, or interruption of antithyroid therapy [1,4]. Obstetric events have also been described as potential triggers, and Graves’ disease remains the most frequent underlying cause [5].

Thyroid storm presents with multisystem involvement. Neurological symptoms may range from agitation to seizures or coma, while cardiovascular manifestations commonly include tachycardia and atrial fibrillation, sometimes complicated by heart failure or thromboembolic events [6-8]. Gastrointestinal and hepatic symptoms may also occur [1].

Because no single test confirms the diagnosis, thyroid storm remains a clinical diagnosis [4,9]. The Burch-Wartofsky Point Scale, originally described by Burch and Wartofsky, is widely used to support clinical assessment [1], although it may overestimate severity in patients with overlapping conditions [10,11]. Alternatively, the Japanese Thyroid Association criteria emphasize organ dysfunction and may provide greater specificity [5,12]. In practice, both approaches are complementary, and clinical judgment remains essential [9,11].

Management requires early recognition and prompt treatment, often in a critical care setting [12]. Therapy is typically initiated simultaneously and includes beta-blockers, antithyroid drugs, and glucocorticoids, along with treatment of the precipitating factor [1,12]. Although propylthiouracil has traditionally been preferred, recent data suggest comparable outcomes with methimazole [12]. This is particularly relevant in settings where propylthiouracil is not available, such as Mexico.

This case series aims to describe the clinical variability and management of thyroid storm across different presentations, highlighting diagnostic challenges in real-world clinical settings.

## Case presentation

Case 1

A 51-year-old man presented to the emergency department on October 18, 2024, with new-onset generalized tonic-clonic seizures. He had a known history of hyperthyroidism, type 2 diabetes mellitus, and hypertension, all with inadequate follow-up. He had previously been treated with methimazole but discontinued therapy approximately five years earlier and had not been under endocrinology care since that time. Family history was notable for thyroid disease in several first-degree relatives.

According to relatives, symptoms began approximately eight hours prior to admission, when he developed a generalized tonic-clonic seizure at home associated with loss of consciousness and urinary incontinence. He subsequently experienced a total of five seizure episodes, including three at home and two during transport to the hospital. On arrival, he was drowsy and poorly responsive, with a Glasgow Coma Scale score of 11.

Initial vital signs showed a heart rate of 123 beats per minute, blood pressure of 130/90 mmHg, respiratory rate of 24 breaths per minute, temperature of 36.1°C, and oxygen saturation of 97% on room air. Capillary glucose was 143 mg/dL.

On examination, he appeared clinically volume-depleted and had a fine tremor in both hands. The thyroid gland was diffusely enlarged (grade II) without palpable nodules. Cardiac auscultation revealed an irregular rhythm without murmurs. Lung examination was unremarkable. Once his mental status improved, neurological examination showed no focal deficits.

Laboratory results on admission showed hemoglobin 15.56 g/dL, hematocrit 48.94%, mean corpuscular volume 83.16 fL, leukocytes 13.18 × 10³/µL with neutrophilia (10.11 × 10³/µL) and lymphocytes 1.43 × 10³/µL, and platelets 162 × 10³/µL. Serum glucose was 154 mg/dL, urea 56 mg/dL, blood urea nitrogen 26 mg/dL, and creatinine 0.9 mg/dL. Electrolytes showed sodium 141 mmol/L, potassium 3.5 mmol/L, and chloride 106 mmol/L. Liver function tests revealed total protein 7.8 g/dL, albumin 3.7 g/dL, total bilirubin 1.37 mg/dL (direct 0.80 mg/dL, indirect 0.57 mg/dL), aspartate aminotransferase 41 U/L, alanine aminotransferase 31 U/L, and gamma-glutamyl transferase 73 U/L. Coagulation studies showed a prothrombin time of 15.10 seconds, an international normalized ratio (INR) of 1.30, an activated partial thromboplastin time of 36.5 seconds, and fibrinogen of 563 mg/dL. Thyroid function tests revealed a suppressed thyroid-stimulating hormone (TSH) of 0.00 µIU/mL, with elevated total triiodothyronine (T3) of 4.13 and total thyroxine (T4) of 24.24, consistent with thyrotoxicosis. Free T4 was not available at the time of admission due to institutional limitations in urgent testing; however, clinical findings and elevated total T3 and T4 were sufficient to support the diagnosis of thyrotoxicosis in this context.

Laboratory findings are summarized in Table 1.

**Table 1:** Case 1 laboratory findings on admission

Parameter	Value	Reference range
Hemoglobin (g/dL)	15.56	13.5-17.5
Hematocrit (%)	48.94	41-53
Mean corpuscular volume (fL)	83.16	80-100
Leukocytes (×10³/µL)	13.18	4.0-10.0
Neutrophils (×10³/µL)	10.11	1.5-7.5
Lymphocytes (×10³/µL)	1.43	1.0-4.0
Platelets (×10³/µL)	162	150-400
Glucose (mg/dL)	154	70-100
Urea (mg/dL)	56	15-40
Blood urea nitrogen (mg/dL)	26	7-20
Creatinine (mg/dL)	0.9	0.6-1.2
Sodium (mmol/L)	141	135-145
Potassium (mmol/L)	3.5	3.5-5.0
Chloride (mmol/L)	106	98-107
Total protein (g/dL)	7.8	6.0-8.3
Albumin (g/dL)	3.7	3.5-5.0
Aspartate aminotransferase (U/L)	41	10-40
Alanine aminotransferase (U/L)	31	7-56
Total bilirubin (mg/dL)	1.37	0.3-1.2
Direct bilirubin (mg/dL)	0.80	0.0-0.3
Indirect bilirubin (mg/dL)	0.57	0.2-0.8
Gamma-glutamyl transferase (U/L)	73	9-48
Prothrombin time (seconds)	15.10	11-13.5
International normalized ratio	1.30	0.8-1.2
Activated partial thromboplastin time (seconds)	36.5	2-35
Fibrinogen (mg/dL)	563	200-400
Thyroid-stimulating hormone (µIU/mL)	0.00	0.4-4.0
Total triiodothyronine (T3) (ng/mL)	4.13	0.8-2.0
Total thyroxine (T4) (µg/dL)	24.24	5.0-12.0

Urinalysis showed significant hematuria, leukocyturia, and bacteriuria. A repeat sample demonstrated positive nitrites and increased inflammatory cells. Urine culture subsequently grew *Escherichia coli* and *Proteus penneri*, supporting the diagnosis of a urinary tract infection.

**Figure 1:**
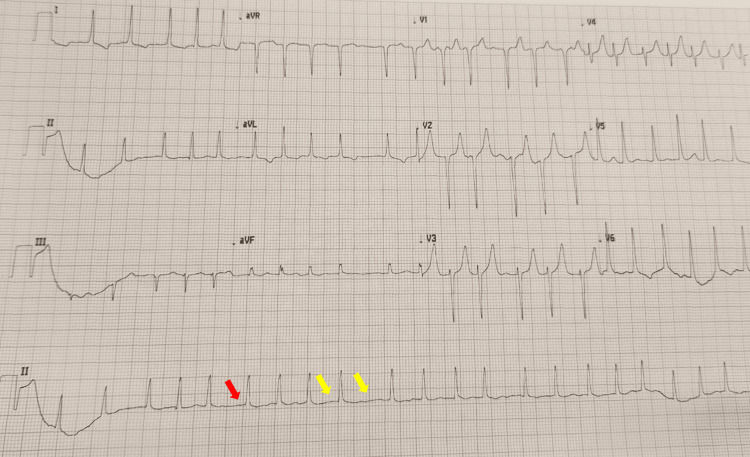
Electrocardiogram showing atrial fibrillation with rapid ventricular response (Case 1) Twelve-lead electrocardiogram demonstrating atrial fibrillation with rapid ventricular response, with an estimated ventricular rate of approximately 140 beats per minute and irregularly irregular R–R intervals (yellow arrows). No discernible P waves are seen (red arrow). No acute ischemic changes are present. Findings are consistent with thyrotoxicosis-associated atrial fibrillation. Standard calibration is 25 mm/s and 10 mm/mV.

**Figure 2:**
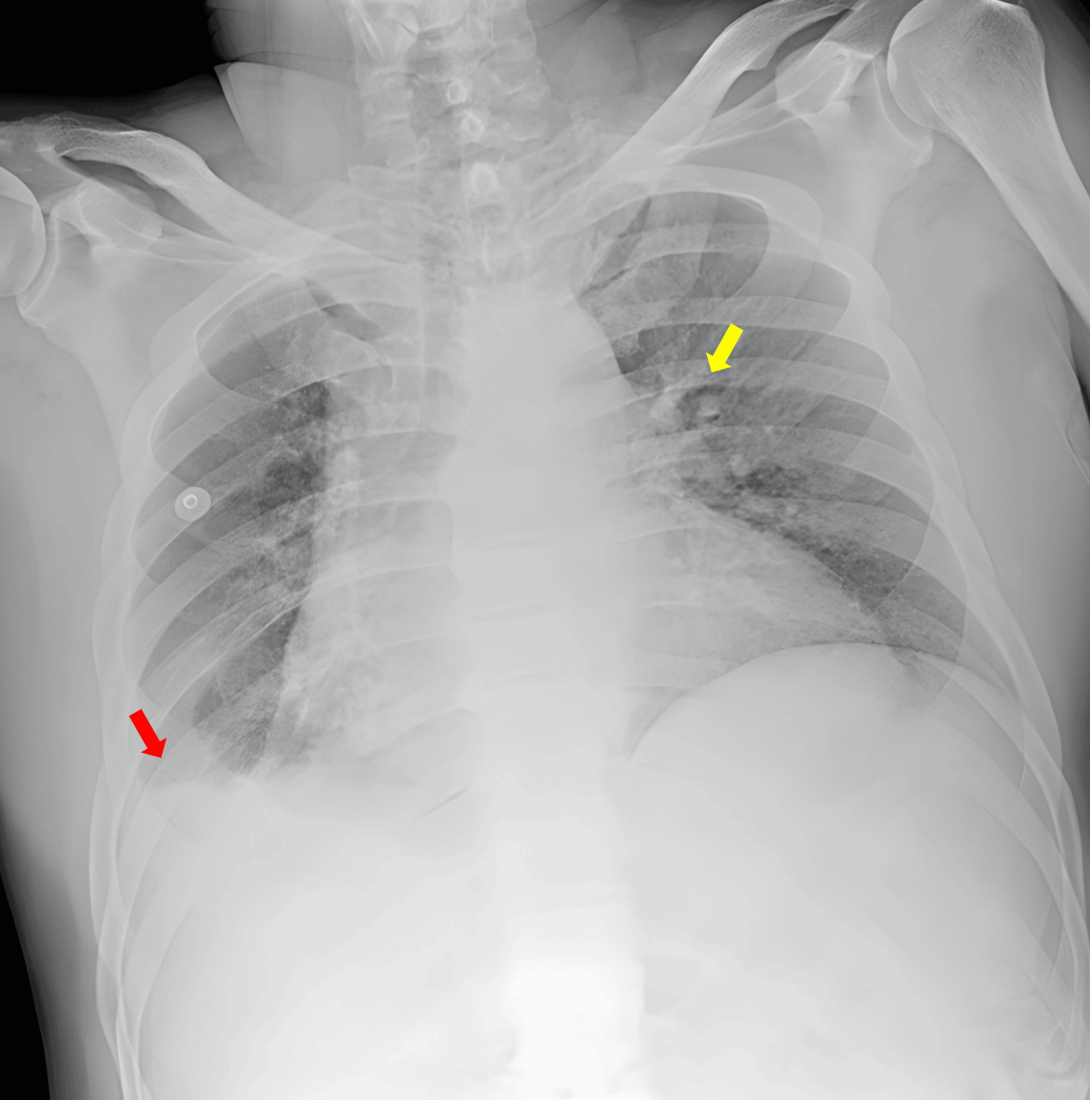
Chest radiograph showing pulmonary vascular congestion and right-sided pleural effusion (Case 1) Chest radiograph demonstrating pulmonary vascular congestion with cephalization of flow (yellow arrow) and a small right-sided pleural effusion with blunting of the right costophrenic angle (red arrow). Findings are consistent with cardiopulmonary involvement in the setting of thyroid storm.

Electrocardiography revealed atrial fibrillation with rapid ventricular response, with an estimated heart rate of approximately 138 beats per minute and irregular R-R intervals (Figure 1). Chest radiography demonstrated increased vascular markings with cephalization of flow and a small right-sided pleural effusion (Figure 2).

Computed tomography of the brain showed no acute findings; however, the original images were not available for inclusion. Bilateral frontal white matter hypodensities were noted, likely reflecting chronic microangiopathic changes.

Based on the overall clinical picture and the presence of a documented precipitating infection, a Burch-Wartofsky score of 65 points was calculated, highly suggestive of thyroid storm. The diagnosis was also consistent with the Japanese Thyroid Association criteria, given the presence of thyrotoxicosis with central nervous system and cardiovascular involvement.

Treatment was initiated promptly with methimazole 20 mg every six hours, propranolol 60 mg every 4-5 hours, and intravenous dexamethasone. He also received intravenous fluids, antipyretics, antiepileptic therapy, and empiric ceftriaxone for the urinary tract infection.

Over the following days, the patient improved clinically. No further seizures occurred, and adequate heart rate control was achieved. Once stable and able to tolerate oral therapy, he was discharged on methimazole 20 mg every six hours, propranolol 80 mg every four hours, and apixaban 5 mg twice daily for atrial fibrillation, with outpatient endocrinology follow-up arranged.

Free T4 was not available at the time of admission; however, the diagnosis of thyrotoxicosis was supported by suppressed TSH and elevated total T3 and T4 in the appropriate clinical context. The underlying etiology of hyperthyroidism could not be determined during hospitalization due to the acute presentation and clinical instability; however, this did not impact initial management, which is guided primarily by the severity of thyrotoxicosis rather than etiology in the acute setting.

Differential diagnoses at presentation included primary seizure disorder, central nervous system infection, metabolic derangements, and acute cerebrovascular events.

Case 2

A 24-year-old woman presented to the emergency department on February 22, 2023, with generalized weakness and lower abdominal pain. She had no prior diagnosis of thyroid disease. Over the preceding three months, she had experienced recurrent episodes of sinus tachycardia and was evaluated by cardiology, including an echocardiogram that was reported as normal. She was not receiving any treatment at that time.

Two days prior to admission, she developed vaginal bleeding with the passage of tissue and a fever up to 41°C. She was initially evaluated at another hospital, where she received misoprostol for presumed incomplete abortion. Serum beta-human chorionic gonadotropin (β-hCG) was reported as <0.10 mIU/mL. She requested voluntary discharge the following day. After discharge, she continued to feel unwell and presented to our institution with persistent generalized weakness and hypogastric abdominal pain radiating to the back.

On arrival, she was markedly tachycardic, with a heart rate of 194 beats per minute, respiratory rate of 15 breaths per minute, oxygen saturation of 99% on room air, and blood pressure of 117/64 mmHg. Given the severity of her presentation, she was admitted to the intensive care unit with suspected thyroid storm.

During her ICU stay, she remained somnolent but arousable, with persistent sinus tachycardia ranging from 101 to 140 beats per minute. She also reported frequent loose stools. She did not require supplemental oxygen and was able to tolerate oral intake. Initial treatment included propranolol 40 mg every six hours, methimazole 20 mg every eight hours, and hydrocortisone 100 mg every six hours, along with intravenous fluids and thromboprophylaxis with enoxaparin.

Following clinical improvement, she was transferred to the general medicine ward. At that time, her heart rate was 96 beats per minute, blood pressure 120/70 mmHg, respiratory rate 21 breaths per minute, temperature 35.1°C, and oxygen saturation 97% on room air. Her weight was 47 kg and height 1.67 m.

On physical examination, she appeared thin and chronically ill. A large diffuse goiter (grade III) was noted on inspection. She also had ophthalmopathy and right-sided strabismus. A fine tremor was present. Cardiac examination revealed a regular rhythm without murmurs. Lung fields were clear. The abdomen was soft and non-tender, without peritoneal signs. There was no peripheral edema.

Further history revealed several months of untreated hyperthyroid symptoms, including unintentional weight loss of approximately 35 kg over three months, increased appetite, heat intolerance, hair loss, tremor, palpitations, exertional dyspnea, and somnolence.

Thyroid function tests obtained on admission showed suppressed thyroid-stimulating hormone (TSH) of 0.00 µIU/mL, free thyroxine (FT4) of 5.26 ng/dL, total triiodothyronine (T3) of 5.23 ng/mL, and total thyroxine (T4) of 15.94 µg/dL, consistent with marked thyrotoxicosis. Additional laboratory evaluation showed glucose 117 mg/dL, urea 45 mg/dL, blood urea nitrogen 21 mg/dL, and creatinine 0.4 mg/dL. Electrolytes showed sodium 140 mmol/L, potassium 3.4 mmol/L, and chloride 108 mmol/L. Venous blood gas analysis showed pH 7.49, pCO₂ 34 mmHg, pO₂ 32 mmHg, bicarbonate 25.9 mmol/L, and lactate 0.9 mmol/L. Liver function tests showed total protein 5.1 g/dL, albumin 2.7 g/dL, total bilirubin 1.0 mg/dL (direct 0.52 mg/dL, indirect 0.48 mg/dL), alanine aminotransferase 22 U/L, aspartate aminotransferase 38 U/L, gamma-glutamyl transferase 48 U/L, and alkaline phosphatase 94 U/L. Complete blood count showed hemoglobin 10.39 g/dL, hematocrit 31.81%, mean corpuscular volume 75 fL, leukocytes 9 × 10³/µL, neutrophils 6.81 × 10³/µL, and platelets 125 × 10³/µL.

Laboratory findings are summarized in Table 2.

**Table 2:** Case 2 laboratory findings on admission

Parameter	Value	Reference range
Thyroid-stimulating hormone (µIU/mL)	0.00	0.4-4.0
Free thyroxine (FT4) (ng/dL)	5.26	0.8-1.8
Total triiodothyronine (T3) (ng/mL)	5.23	0.8-2.0
Total thyroxine (T4) (µg/dL)	15.94	5.0-12.0
Glucose (mg/dL)	117	70-100
Urea (mg/dL)	45	15-40
Blood urea nitrogen (mg/dL)	21	7-20
Creatinine (mg/dL)	0.4	0.6-1.2
Sodium (mmol/L)	140	135-145
Potassium (mmol/L)	3.4	3.5-5.0
Chloride (mmol/L)	108	98-107
pH	7.49	7.35-7.45
pCO₂ (mmHg)	34	35-45
pO₂ (mmHg)	32	30-40 (venous)
HCO₃ (mmol/L)	25.9	22-28
Lactate (mmol/L)	0.9	0.5-2.0
Total protein (g/dL)	5.1	6.0-8.3
Albumin (g/dL)	2.7	3.5-5.0
Total bilirubin (mg/dL)	1.0	0.3-1.2
Direct bilirubin (mg/dL)	0.52	0.0-0.3
Indirect bilirubin (mg/dL)	0.48	0.2-0.8
Alanine aminotransferase (U/L)	22	7-56
Aspartate aminotransferase (U/L)	38	10-40
Gamma-glutamyl transferase (U/L)	48	9-48
Alkaline phosphatase (U/L)	94	44-147
Hemoglobin (g/dL)	10.39	12-16
Hematocrit (%)	31.81	36-46
Mean corpuscular volume (fL)	75	80-100
Leukocytes (×10³/µL)	9	4.0-10.0
Neutrophils (×10³/µL)	6.81	1.5-7.5
Platelets (×10³/µL)	125	150-400

Computed tomography of the abdomen showed free fluid in multiple abdominal compartments, including perihepatic, perivesicular, perisplenic, paracolic, interloop, and pelvic regions. Bilateral pleural effusions were also noted, more prominent on the left side, associated with passive atelectasis in both lower lobes. The liver appeared normal in size but with decreased parenchymal density, without focal lesions. The remaining abdominal organs were unremarkable.

Transvaginal ultrasound showed a uterus measuring 5.2 × 3.2 cm with an endometrial thickness of 13 mm, a right adnexal cystic lesion of 1.8 cm, and moderate free fluid in the posterior cul-de-sac.

The electrocardiogram showed a sinus rhythm with a heart rate of 120 beats per minute, QRS duration of 120 ms, normal axis at 71°, and no evidence of ischemia or conduction abnormalities. Imaging findings were obtained from official radiology reports (not shown).

Based on the clinical findings, a Burch-Wartofsky score of 95 points was calculated, consistent with severe thyroid storm.

Treatment was continued with antithyroid therapy, beta-blockers, and corticosteroids. Because propylthiouracil was not consistently available, methimazole was continued and later adjusted to 20 mg every four hours. Propranolol was increased to 60 mg every six hours due to persistent tachycardia.

During hospitalization, the patient showed gradual clinical improvement, with better heart rate control, resolution of abdominal pain, and overall clinical stabilization. She was able to ambulate and tolerate oral intake without difficulty.

Differential diagnoses included septic abortion, pelvic inflammatory disease, acute abdomen, and systemic infection with associated hemodynamic instability.

Case 3

A 45-year-old woman presented to the emergency department on March 30, 2025, with fever and progressive neurological deterioration. Her past medical history was significant for long-standing, untreated hyperthyroidism, hypertension without regular follow-up, and a recent ischemic stroke in February 2025 with residual functional impairment (modified Rankin score 3). She had poor adherence to medical therapy.

According to her daughter, symptoms began two days prior to admission with unquantified fever, followed by progressive somnolence and slurred speech one day before presentation. She was initially evaluated at a local clinic and referred to our institution with concern for recurrent cerebrovascular events.

On arrival, her vital signs showed a heart rate of 84 beats per minute, blood pressure of 153/88 mmHg, respiratory rate of 20 breaths per minute, temperature of 36.8°C, and oxygen saturation of 94% on room air. She was admitted for further evaluation and management.

On physical examination, she appeared chronically ill and cachectic. Neurologically, she was somnolent with dysarthria and limited cooperation. She had right-sided upper motor neuron facial weakness with forehead sparing, along with asymmetric motor deficits: strength was 2/5 in the right upper extremity, 4/5 in the right lower extremity, and preserved strength on the left side. Tone was decreased on the right hemibody, and reflexes were asymmetric. There were no meningeal signs.

Examination of the neck revealed a diffusely enlarged thyroid gland, firm on palpation and without nodules. Cardiac examination revealed an irregular rhythm without murmurs.

Laboratory evaluation demonstrated hemoglobin 10.50 g/dL, hematocrit 33%, mean corpuscular volume 80.94 fL, mean corpuscular hemoglobin 25.76 pg, leukocytes 8.14 × 10³/µL, neutrophils 5.80 × 10³/µL, lymphocytes 1.16 × 10³/µL, and platelets 135 × 10³/µL. The metabolic panel showed glucose 60 mg/dL, urea 26 mg/dL, blood urea nitrogen 12 mg/dL, creatinine 0.4 mg/dL, sodium 137 mmol/L, potassium 3.5 mmol/L, and chloride 107 mmol/L. Venous blood gas analysis showed pH 7.36, pCO₂ 34 mmHg, pO₂ 23 mmHg, bicarbonate 20.4 mmol/L, and lactate 3.3 mmol/L. Lactate dehydrogenase was 175 U/L.

Liver function tests showed total protein 7.3 g/dL, albumin 3.3 g/dL, total bilirubin 0.67 mg/dL (direct 0.39 mg/dL, indirect 0.28 mg/dL), alanine aminotransferase 34 U/L, aspartate aminotransferase 46 U/L, gamma-glutamyl transferase 62 U/L, and alkaline phosphatase 113 U/L. Coagulation studies showed a prothrombin time of 13 seconds, INR 1.12, activated partial thromboplastin time 32.9 seconds, and fibrinogen 436 mg/dL. Cardiac biomarkers were elevated, including troponin 39.7 ng/L, creatine phosphokinase 26 U/L, and CK-MB 31 U/L.

Thyroid function tests showed a suppressed TSH of 0.00 µIU/mL, FT4 of 5.14 ng/dL, T3 of 2.15 ng/mL, and T4 of 20 µg/dL, consistent with uncontrolled thyrotoxicosis.

Laboratory findings are summarized in Table 3.

**Table 3:** Case 3 laboratory findings on admission

Parameter	Value	Reference range
Hemoglobin (g/dL)	10.50	12-16
Hematocrit (%)	33	36-46
Mean corpuscular volume (fL)	80.94	80-100
Mean corpuscular hemoglobin (pg)	25.76	27-33
Leukocytes (×10³/µL)	8.14	4.0-10.0
Neutrophils (×10³/µL)	5.80	1.5-7.5
Lymphocytes (×10³/µL)	1.16	1.0-4.0
Platelets (×10³/µL)	135	150-400
Glucose (mg/dL)	60	70-100
Urea (mg/dL)	26	15-40
Blood urea nitrogen (mg/dL)	12	7-20
Creatinine (mg/dL)	0.4	0.6-1.2
Sodium (mmol/L)	137	135-145
Potassium (mmol/L)	3.5	3.5-5.0
Chloride (mmol/L)	107	98-107
Lactate dehydrogenase (U/L)	175	140-280
pH	7.36	7.35-7.45
pCO₂ (mmHg)	34	35-45
pO₂ (mmHg)	23	30-40 (venous)
HCO₃ (mmol/L)	20.4	22-28
Lactate (mmol/L)	3.3	0.5-2.0
Troponin (ng/L)	39.7	<14
Creatine phosphokinase (U/L)	26	30-200
CK-MB (U/L)	31	<5
Total protein (g/dL)	7.3	6.0-8.3
Albumin (g/dL)	3.3	3.5-5.0
Total bilirubin (mg/dL)	0.67	0.3-1.2
Direct bilirubin (mg/dL)	0.39	0.0-0.3
Indirect bilirubin (mg/dL)	0.28	0.2-0.8
Alanine aminotransferase (U/L)	34	7-56
Aspartate aminotransferase (U/L)	46	10-40
Gamma-glutamyl transferase (U/L)	62	9-48
Alkaline phosphatase (U/L)	113	44-147
Prothrombin time (seconds)	13	11-13.5
International normalized ratio	1.12	0.8-1.2
Activated partial thromboplastin time (seconds)	32.9	25-35
Fibrinogen (mg/dL)	436	200-400
Free thyroxine (FT4) (ng/dL)	5.14	0.8-1.8
Triiodothyronine (T3) (ng/mL)	2.15	0.8-2.0
Total thyroxine (T4) (µg/dL)	20	5.0-12.0
Thyroid-stimulating hormone (µIU/mL)	0.00	0.4-4.0

Computed tomography of the brain demonstrated an acute-to-subacute ischemic infarction in the right frontal and temporal lobes within the middle cerebral artery territory, along with chronic infarcts in the occipital and frontal regions.

**Figure 3:**
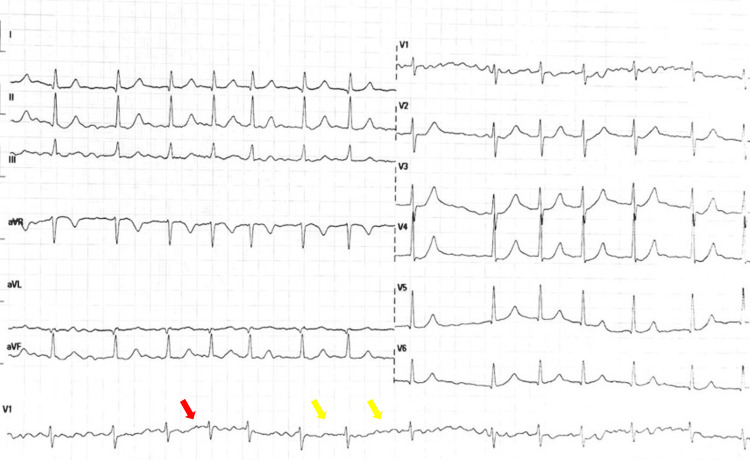
Electrocardiogram showing atrial fibrillation (Case 3) Twelve-lead electrocardiogram demonstrating atrial fibrillation with irregularly irregular R-R intervals (yellow arrows). No discernible P waves are observed, with a fibrillatory baseline visible in lead V1 (red arrow). No acute ischemic changes are present. Standard calibration is 25 mm/s and 10 mm/mV.

Electrocardiography revealed atrial fibrillation with an irregularly irregular rhythm (Figure 3).

Given the clinical presentation, a Burch-Wartofsky score of 40 points was calculated, consistent with impending thyroid storm. The likely precipitating factor was infection, supported by findings of a urinary tract infection due to extended-spectrum beta-lactamase (ESBL)-producing *E. coli*.

Once thyroid storm was suspected, the patient was started on high-dose methimazole (20 mg every four hours) and propranolol (60 mg every six hours) during the acute phase, along with anticoagulation and antibiotic therapy. She initially received ceftriaxone and clarithromycin, with subsequent adjustment based on microbiological results.

During hospitalization, the patient showed gradual clinical improvement. Her mental status improved, and she became alert and oriented. She remained hemodynamically stable without further neurological deterioration. After completing antibiotic therapy and achieving clinical stabilization, she was discharged on methimazole 20 mg every 12 hours (administered as four 5 mg tablets twice daily) and propranolol 40 mg every 12 hours, with plans for outpatient endocrinology follow-up and consideration of definitive therapy.

Differential diagnoses included recurrent ischemic stroke, central nervous system infection, metabolic encephalopathy, and sepsis-related neurological dysfunction.

## Discussion

Thyroid storm remains a challenging condition to recognize in clinical practice, despite being well described in the literature. One of the main difficulties is that it does not always present in a predictable manner. In our series, the three patients demonstrated markedly different clinical patterns, including predominant neurological involvement with seizures, an obstetric-associated systemic deterioration, and cardiovascular and thromboembolic complications in the setting of atrial fibrillation and ischemic stroke. This variability has been consistently described in previous reports, where thyroid storm behaves more like a multisystem syndrome than a single, uniform entity [1,2].

In all cases, a precipitating factor was identified. Two patients had evidence of infection, while in the second case, the clinical picture developed after an obstetric event. These findings are consistent with prior reports in which infections represent the most common trigger, followed by other stressors such as surgery, trauma, or interruption of antithyroid therapy [1,3,4]. In practice, identifying and treating the precipitating factor is essential, as it often contributes significantly to clinical deterioration and influences outcomes [5,6].

Thyroid storm represents an extreme manifestation of thyrotoxicosis in which clinical severity is not solely determined by circulating hormone levels but rather by the systemic response to thyroid hormone excess. This exaggerated response is thought to involve increased adrenergic activity, enhanced peripheral conversion of thyroid hormones, and differential tissue sensitivity. These mechanisms may help explain atypical manifestations such as seizures, obstetric deterioration, or cerebrovascular events, which are increasingly recognized as part of the heterogeneous clinical spectrum of thyroid storm [3].

To better illustrate this variability, a comparative summary of the cases is presented in Table 4. Case 1 was notable for neurological manifestations with seizures and an infectious trigger, Case 2 demonstrated the highest severity with systemic decompensation in the context of an obstetric event, and Case 3 presented with cardiovascular and neurological complications in a patient with prior cerebrovascular disease. Despite differences in clinical presentation, all patients shared multisystem involvement and responded to similar therapeutic strategies, highlighting the importance of early recognition and standardized management.

**Table 4:** Comparative clinical characteristics of the three patients in the present case series ESBL: extended-spectrum beta-lactamase, ICU: intensive care unit.

Parameter	Case 1	Case 2	Case 3
Age (years)	51	24	45
Sex	Male	Female	Female
Underlying thyroid disease	Known hyperthyroidism, untreated	Undiagnosed hyperthyroidism	Known hyperthyroidism, untreated
Precipitating factor	Urinary tract infection	Obstetric event (incomplete abortion)	Urinary tract infection (ESBL-producing *E. coli*)
Key distinguishing feature	Seizures	Obstetric trigger with systemic deterioration	Ischemic stroke
Neurological involvement	Seizures, altered consciousness	Somnolence	Stroke, somnolence, focal deficits
Cardiovascular findings	Atrial fibrillation with rapid ventricular response	Severe sinus tachycardia	Atrial fibrillation
Maximum temperature	36.1°C	41°C	36.8°C
Burch-Wartofsky score	65	95	40
ICU admission	No	Yes	No
Antithyroid therapy	Methimazole	Methimazole	Methimazole
Adjunctive therapy	Propranolol, corticosteroids	Propranolol, corticosteroids	Propranolol, corticosteroids
Outcome	Clinical improvement with stabilization at discharge	Clinical improvement with stabilization at discharge	Clinical improvement with stabilization at discharge

From a diagnostic perspective, our cases reflect both the usefulness and the limitations of the Burch-Wartofsky Point Scale. In the first two patients, the score aligned well with the clinical impression. However, in the third case, the score suggested impending thyroid storm despite significant clinical deterioration, largely due to overlapping neurological disease. This highlights a known limitation of the scale, as it may be influenced by comorbid conditions that resemble or overlap with thyroid storm [7,8]. The Burch-Wartofsky Point Scale is generally considered more sensitive but less specific, which may lead to overestimation of severity in patients with overlapping conditions. In contrast, the Japanese Thyroid Association criteria, which emphasize organ dysfunction, tend to be more specific but may miss milder or atypical cases [4,6]. In practice, both approaches are complementary, and clinical judgment remains essential.

Cardiovascular involvement was a recurrent finding in our patients. Atrial fibrillation was present in two cases, and in one patient, it was associated with ischemic stroke. Thyroid hormone excess is known to increase cardiac excitability and predispose to arrhythmias, particularly atrial fibrillation, which may increase the risk of thromboembolic complications [9,10]. These findings underscore the importance of early heart rate control and appropriate anticoagulation when indicated.

Regarding treatment, all patients were managed with beta-blockers, corticosteroids, and antithyroid therapy with methimazole. Iodine therapy was not administered due to institutional practice patterns and limited availability. Although propylthiouracil has traditionally been preferred due to its effect on peripheral conversion of T4 to T3, its availability is limited in certain settings, including our institution. Recent observational data suggest that outcomes may be comparable between propylthiouracil and methimazole in critically ill patients with thyroid storm, supporting the use of methimazole as a reasonable therapeutic option within the limitations of this case series [12]. The use of glucocorticoids may also have contributed to clinical improvement through inhibition of peripheral hormone conversion and modulation of the stress response [1,12].

Another important observation is that the severity of clinical presentation did not correlate closely with thyroid hormone levels. Instead, neurological and cardiovascular involvement appeared to better reflect the degree of illness. This reinforces the concept that thyroid storm represents a dysregulated systemic response rather than a condition defined solely by biochemical thresholds [2].

These cases highlight how thyroid storm can overlap with other acute conditions and present with atypical features. For this reason, it should remain in the differential diagnosis whenever unexplained systemic deterioration develops in a patient with known or suspected thyrotoxicosis. While scoring systems are useful, they should always be interpreted within the broader clinical context.

In resource-limited settings, treatment strategies may vary depending on medication availability; however, this should not delay prompt initiation of therapy, as early treatment remains critical to reducing morbidity and mortality.

This case series has several limitations. As a small, single-center study, the findings may not be generalizable to broader populations. Additionally, the retrospective nature limits causal inference. Some diagnostic data, including complete thyroid profiles and imaging studies, were not consistently available due to the acute clinical setting. Furthermore, the absence of long-term follow-up restricts assessment of outcomes beyond hospitalization. Despite these limitations, the cases provide clinically relevant insights into the recognition and management of thyroid storm in real-world settings.

## Conclusions

Thyroid storm can be difficult to recognize, especially when the presentation is not typical. In our series, the diagnosis was challenged by neurological, obstetric, and cardiovascular manifestations, highlighting the wide clinical variability of this condition. The main message is that clinical suspicion remains essential, particularly when a patient with known or suspected thyrotoxicosis deteriorates without a clear explanation. Our cases suggest that methimazole may be considered a reasonable therapeutic option in this context, particularly where propylthiouracil is not available, although interpretation is limited by the small sample size.
